# Characterization of Wheat Varieties Using Terahertz Time-Domain Spectroscopy

**DOI:** 10.3390/s150612560

**Published:** 2015-05-27

**Authors:** Hongyi Ge, Yuying Jiang, Feiyu Lian, Yuan Zhang, Shanhong Xia

**Affiliations:** 1 State Key Laboratory of Transducer Technology, Institute of Electronics, Chinese Academy of Sciences, Beijing 100080, China; E-Mails: jiangyuying11@163.com (Y.J.); shxia@mail.ie.ac.cn (S.X.); 2 University of the Chinese Academy of Sciences, Beijing 100080, China; 3 Key Laboratory of Grain Information Processing & Control, Ministry of Education, Zhengzhou 450001, China; E-Mails: lfywork@163.com (F.L.); zhangyuan@haut.edu.cn (Y.Z.)

**Keywords:** terahertz time-domain spectroscopy, wheat varieties, absorption spectrum, interval partial least squares

## Abstract

Terahertz (THz) spectroscopy and multivariate data analysis were explored to discriminate eight wheat varieties. The absorption spectra were measured using THz time-domain spectroscopy from 0.2 to 2.0 THz. Using partial least squares (PLS), a regression model for discriminating wheat varieties was developed. The coefficient of correlation in cross validation (R) and root-mean-square error of cross validation (RMSECV) were 0.985 and 1.162, respectively. In addition, interval PLS was applied to optimize the models by selecting the most appropriate regions in the spectra, improving the prediction accuracy (*R* = 0.992 and RMSECV = 0.967). Results demonstrate that THz spectroscopy combined with multivariate analysis can provide rapid, nondestructive discrimination of wheat varieties.

## 1. Introduction

Wheat is one of the most important agricultural products in the world. Due to increasing free trade, wheat varieties from diverse origins are widely available in the markets. However, the nutrition and processing quality of wheat varieties differ, and false wheat seeds can cause great losses for farmers. Thus, there is a demand for rapid analytical methods to discriminate the material properties of wheat. Traditional methods such as morphology analysis, physics, chemical methods, machine vision, and DNA molecular marker analysis enable sensitive classification of wheat varieties [1,2,3,4]. However, these methods are time-consuming and require complex operation. Spectroscopic methods such as near infrared, mid-infrared, and Raman spectroscopy are adequate analytical tools that have been used largely for material classification [5,6,7]. However, few studies have utilized the far infrared or terahertz (THz) band for discriminating wheat varieties.

Terahertz radiation, which occupies frequencies between 0.1 and 10 THz, lies between microwaves and infrared bands in the electromagnetic spectrum. Due to the rapid advances in generating, detecting, and analyzing THz radiation, THz spectroscopy has been recently developed as an analytical method [8,9]. Because many molecules have unique spectral fingerprints in THz band as a result of the vibrational transitions of the molecules, THz spectroscopy can be used for discriminating materials. Furthermore, THz radiation is non-destructive and can penetrate many nonpolar materials. As a result, THz spectroscopy has been successfully applied in detecting explosives [10] and drugs [11], in the field of biological sciences [12], and in food safety control [13].

The aim of the present study is to investigate the potential of THz spectroscopy as a non-destructive method to discriminate wheat varieties. The THz spectra of wheat varieties were measured and analyzed in the frequency of 0.2–2 THz. In addition, chemometric methods were used to evaluate wheat varieties based on THz spectra. The partial least squares (PLS) and interval PLS (iPLS) methods were used to obtain better discrimination results.

## 2. Materials and Methods

### 2.1. Experimental Setup

A conventional terahertz transmission spectroscopy system was used in the experiment. The mode-locked Ti-sapphire femtosecond laser, which provided 100-fs pulses at a wavelength of 800 nm and a repeating frequency of 80 MHz, was divided into two beams (pump beam and probe beam) using a polarization beam splitter (PBS). The THz pulses were generated from the low-temperature-grown GaAs photoconductive antenna with an attached silicon hyperhemispherical lens. The THz radiation from the emitter was collected and focused on the sample by a pair of parabolic mirrors (PM). Electro-optic (EO) detection was employed to observe the THz signal. The transmitted THz radiation was focused and collimated by PM onto the ZnTe EO detector crystal. A detailed description of THz-TDS can be found in Ref. [14]. The THz beam path was filled with nitrogen gas to remove absorption of atmospheric water vapor [15]. The samples were placed at the focal point of the THz beam spectroscope, and the measurements were performed at an ambient temperature of 294 K with a relative humidity of approximately 3%.

Using THz-TDS, we can measure both the phase and amplitude of the THz pulses propagating through the sample and reference (nitrogen gas). A reference pulse signal, *E_ref_*(t), in the absence of wheat and a sample pulse signal, *E_sam_*(t), are recorded. Comparing the sample pulse and reference pulse using a fast Fourier transform, the complex refractive index *N*(ω) can be expressed as follows:

(1) N(ω)=n(ω)−ik(ω)

where *n*(ω) and *k*(ω) are the real refractive index and extinction coefficient, respectively, describing the dispersion and absorption characteristics of the sample. Here, ω is the cyclic frequency, and *i* is the imaginary unit. The complex transmittance function *H*(ω) of sample is given by [16,17,18]:

(2) $$H(\text{ω})=\frac{E_{sam}(\text{ω})}{E_{ref}(\text{ω})}=\frac{4N}{{\left(N+1\right)}^{2}}\exp(\frac{i\text{ω}(N-1)d}{c})=\text{ρ}(\text{ω})\exp(i\text{ϕ}(\text{ω}))$$

where *E_sam_*(ω) and *E_ref_*(ω) are the complex amplitudes of the Fourier transform of *E_ref_*(t) and *E_sam_*(t), respectively, *c* is the speed of light, and ρ(ω) and ϕ(ω) are the amplitude ratio and related phase difference of the reference and sample, respectively.

The *n*(ω) and absorption coefficient *a*(ω) were obtained by:

(3) $$n(\text{ω})=\frac{\text{ϕ}(\text{ω})}{\text{ω}d}c+1$$

(4) $$a(\text{ω})=\frac{2}{d}\ln\left(\frac{4n(\text{ω})}{\rho (\text{ω}){\left[n(\text{ω})+1\right]}^{2}}\right)$$

### 2.2. Sample Preparation

In this study, eight wheat varieties were prepared for the analysis. Samples were supplied by Henan University of Technology, Zhengzhou, China, and were harvested in 2013. The collection of samples is as diverse as possible and representative of the main production areas. These wheat grains are mixtures with different components and complex structures and can have different chemical and physical properties. The sample properties are shown in Table 1.

**Table 1:** The Properties of the eight wheat varieties under consideration.

No.	Wheat Variety	Bulk Density (g/L)	Crude Protein Content (%)	Water Content (%)	Imperfect Grain (%)	Gluten Content (%)
1	Zhengmai 9023	756	14.6	12.5	3.2	27.9
2	Zhouyuan 9369	790	14.9	12.5	3.4	33.0
3	Aobiao	845	14.0	11.5	1.0	26.0
4	DNS	840	14.5	11.9	1.6	38.0
5	Jiamai	830	13.8	12.2	1.8	39.0
6	Jinan 17 wheat	773	15.6	11.5	3.0	34.0
7	Zhoumai 27	798	13.2	12.1	3.8	33.0
8	Yunong 416	787	14.3	12.5	4.0	32.5

The eight wheat samples are identified as Zhengmai 9023, Zhouyuan 9369, Aobiao, DNS, Jiamai, Jinan17 wheat, Zhoumai 27, and Yunong 416 wheat. For each variety of wheat, 20 samples were prepared without further purification before grinding. Generally, the wheat samples can transform to more stable form during the storage and manufacture process, such as grinding and compaction. To form thin, circular slices samples, wheat samples were ground into fine powder for 2 min, which was subsequently sieved by filtering laws using 200-eye sieves; then, the sieved powder was pressed into pellets with a thickness of approximately 1 mm and a diameter of 13 mm under a pressure of 5 tons for 5 min. All sample preparation processes were implemented at room temperature.

### 2.3. Chemometrics Methods

A chemometric analysis was performed to investigate the THz spectral data of wheat varieties using a partial least squares (PLS) regression. The PLS regression is based on latent variables, which are constructed to identify the maximal covariance between two matrices [19]. In particular, PLS was applied to find the best correlation between the spectral data *X* and measured parameter designating the class of interest *Y*. The model is formulated as follows:

(5) $$X=TP^{\text{T}}+E$$

(6) $$Y=UQ^{\text{T}}+F$$

where *X* and *Y* are the input and output matrices, *T* and *U* are the score matrices, *P* and *Q* are the loading matrices that can be regarded as the covariance between *X* and *Y* and between *Y* and *U*, respectively, and *E* and *F* are the residual matrices. In this paper, the absorption coefficient and refractive index of a selected frequency range are used as the input matrix *X*, while the wheat varieties of samples are used as the output matrix *Y*, which can be regarded as the number for each wheat variety sample, and the corresponding wheat variety can be described using number 1–8 given in Table 1.

The interval partial least squares (iPLS) method is a variable selection technique [20] used for identifying the important spectral regions and removing interference from other regions. The iPLS method divides the whole spectrum into subintervals of equal width and develops the PLS model for each subinterval spectrum. The prediction performance of PLS and iPLS is compared by the root-mean-square error of cross-validation (RMSECV) values, and the best subinterval can be selected by the lowest model RMSECV [21]. The quality of the calibration model is evaluated using the correlation coefficient between the reference and predicted value (R), the root-mean-square error (RMSE) of the calibration set (RMSEC), and the root mean square error of the prediction set (RMSEP) [22]. A better model has a better prediction accuracy that provides higher R and lower RMSECV values. The parameters are calculated as follows:

(7) $$RMSECV=\sqrt{\frac{1}{n}\sum_{i=1}^{n}{\left(y_{r}{}^{i}-y_{p}{}^{i}\right)}^{2}}$$

(8) $$R=\frac{\sum_{i=1}^{n}\left(y_{r}{}^{i}-\bar{y_{r}})(y_{p}{}^{i}-\bar{y_{p}}\right)}{\sqrt{\sum_{i=1}^{n}{\left(y_{r}{}^{i}-\bar{y_{r}}\right)}^{2}\sum_{i=1}^{n}{\left(y_{p}{}^{i}-\bar{y_{p}}\right)}^{2}}}$$

where *n* is the number of samples in the calibration sample set, $y_{r}^{i}$ is the reference value of the ith sample, $y_{p}^{i}$ is the predicted value of the ith sample, $\bar{y_{r}}$ is the average of the sample reference values, and $\bar{y_{p}}$ is the average of the predicted values of samples.

## 3. Results and Discussion

### 3.1. Spectra of Wheat Samples

**Figure 1:**
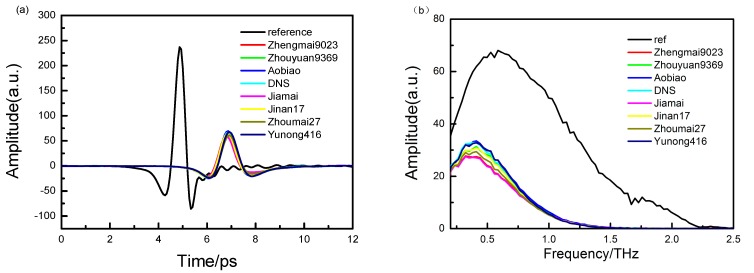
(**a**) Time-domain THz spectra of the eight wheat samples and reference; and (**b**) the frequency spectra of the eight wheat samples and the reference in the range of 0.2–2.5 THz.

**Figure 2:**
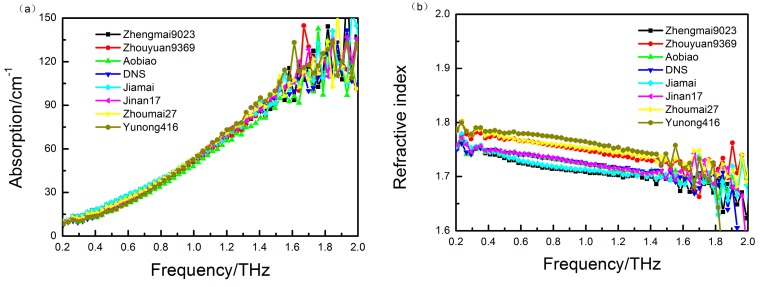
(**a**) The absorption coefficient and (**b**) refractive index for the eight wheat samples in the range of 0.2–2.0 THz.

To remove the random error and increase the signal-to-noise ratio (SNR), each sample is measured three times; the sample spectrum was the average of three scanning spectra in the range of 0.2–2.5 THz, and the reference was measured between every three samples. Figure 1a,b show the time-domain spectra of the eight wheat samples and the corresponding frequency-domain spectra obtained using a fast Fourier transform algorithm. Furthermore, the refractive indices and the absorption coefficients of the eight wheat samples are calculated using Equations (3) and (4), respectively, and the calculated results are shown in Figure 2a,b.

As shown in Figure 1, the effective measurement range is 0.2–2.5 THz, and the sample spectra have narrow bandwidths. Although there is a minor difference in the shift among the samples, there is remarkable shift between the samples and the reference, which indicates that the refractive indices of the samples are different. Moreover, the amplitude changes of the samples indicate that the sample absorption coefficients differ.

**Figure 3:**
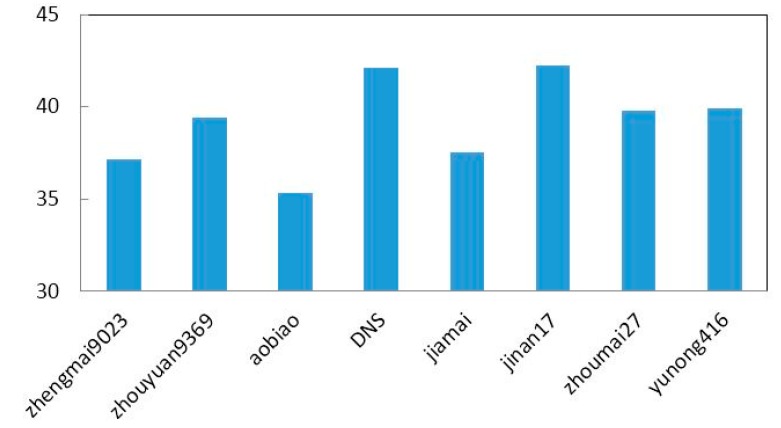
The average absorption coefficient of eight wheat samples.

Because of the similar components in the samples and the chemical complexity of wheat, the optical parameters of the measured wheat samples are very similar, as shown in Figure 2. In order to represent the variation in measurement effectively, the average absorption coefficients of 20 samples of eight wheat samples are shown in Figure 3. The average absorption coefficients are 37.1492 (zhengmai9023), 39.4354 (zhouyuan9369), 35.3358 (aobiao), 42.1133 (DNS), 37.5468 (jiamai), 42.2520 (jinan17), 39.8226 (zhoumai27) and 39.9409 (yunong416), respectively. In addition, the spectra of wheat samples at higher frequencies (above 1.5 THz) produce lower SNR due to the limitation of the dynamic range of the measurement system. Because there are no obvious absorption peaks in the spectra and because the differences in the absorption spectra for the eight wheat samples are not significant, we employ PLS regression to investigate the relationship between the minor spectral differences and the measured wheat varietal properties.

### 3.2. PLS Analysis

The PLS analysis was performed on THz spectral data with the MATLAB software package (Version 2012a, Mathworks Inc., Natick, MA, USA) using the PLS toolbox (Version 4.0, Eigenvector Research Inc., Wenatchee, USA) and user-written scripts. The original spectra with 128 frequency variables were analyzed to establish the correlation between the spectral data and relevant wheat varieties. The eight wheat samples were set as output variables using numbers 1 through 8 (Table 1). The PLS calibration model was developed using a leave-one-out cross-validation calculation. In this study, both the absorption spectra and the refractive spectra of wheat samples from 0.2 to 1.5 THz were applied to obtain the best model. Then, the models were employed to predict the eight varieties of samples. A set of eight varieties of wheat (160 samples in total) was used in this experiment. All the samples were divided into two sets randomly, the calibration set (96 samples) and the prediction set (64 samples). The performance of the cross-validation models is shown in Table 2.

**Table 2:** Calibration and validation results obtained with the effective spectrum PLS Model.

Input Variable	Frequency Range (THz)	Factors	Calibration		Cross Validation	
			R	RMSEC	R	RMSECV
Absorption coefficient	0.2–1.5 THz	5	0.987	0.759	0.983	1.028
Refractive index	0.2–1.5 THz	5	0.982	1.472	0.979	1.684

The absorption coefficient, which has a higher R-value and lower RMSE and RMSECV values, demonstrates better performance than the refractive index for the full-spectrum PLS model. Thus, this indicates that the absorption spectrum-based PLS model is a better model for prediction of wheat varieties compared to the refractive index-based PLS model in the frequency range of 0.2–1.5 THz.

**Figure 4:**
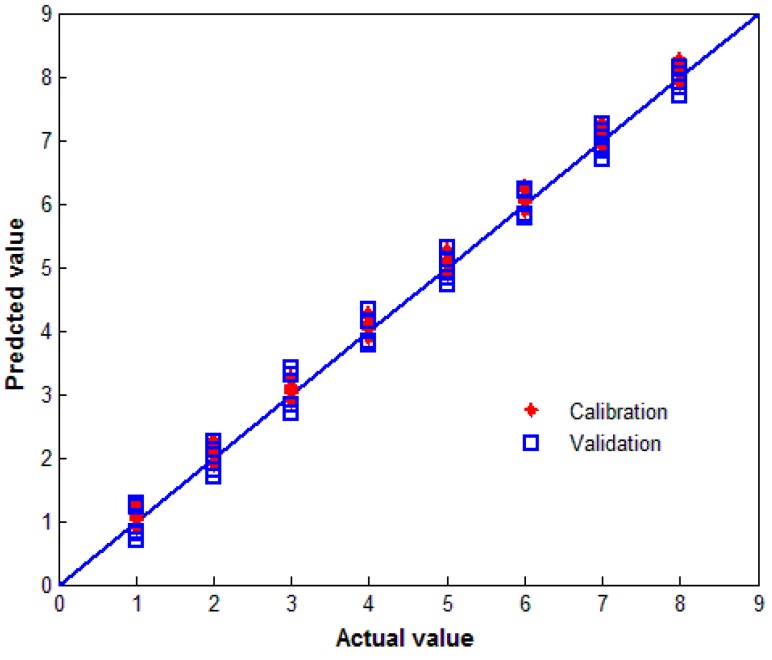
The calibration and validation results for wheat discrimination using the PLS model.

Figure 4 shows the calibration and validation results for wheat varietal discrimination using the absorption spectrum PLS regression model. In the model, the reference line indicates the zero residuals between the predicted and the actual values. Figure 4 shows that the predicted values for all varieties of samples agree with the actual values, indicating that the PLS model can identify wheat varieties.

### 3.3. iPLS Analysis

**Figure 5:**
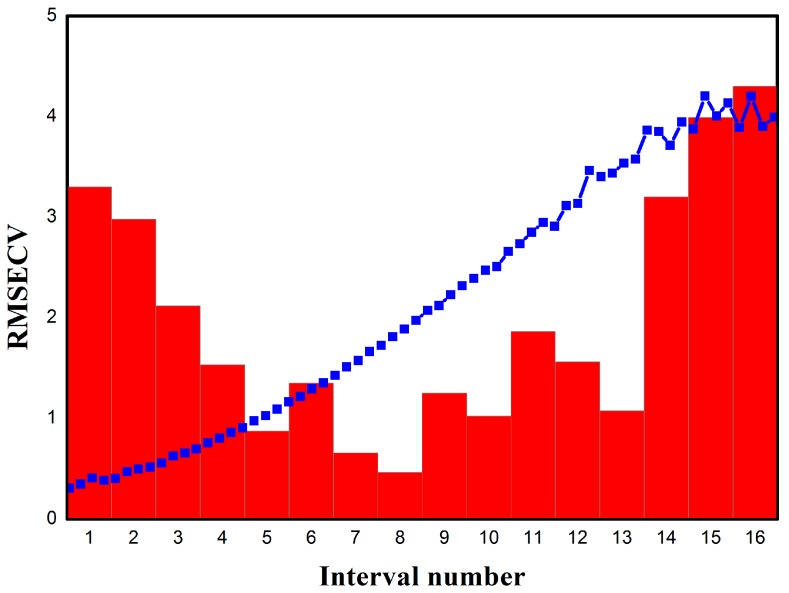
The iPLS results for the THz spectra data. The columns indicate the RMSECV in each subinterval, and the mean absorption spectrum of the wheat samples is overlaid on the plot.

For comparison, iPLS analysis was also performed using the same absorption spectral data sets to improve the model performance. First, the full spectrum was divided into 16 equal subintervals with eight variables. Calibration models were developed for each of the 16 intervals. Then, cross-validation was performed for each of the 16 models. Figure 5 presents the iPLS variable selection results for the discrimination of wheat varieties.

From Figure 5, the width of each column is the same, while the height of each column indicates the RMSECV value calculated by the subinterval PLS model. The eighth column with the interval 57–64, corresponding to the frequency range of 787.5–900 GHz, shows the lowest RMSECV and is selected for developing the model. Therefore, the resulting model should have more precision in discriminating the wheat varieties.

In addition, iPLS selects the most relevant part of the spectrum, which can improve the performance of the model by removing the noise and interference from other regions. In the experiment, a different number of intervals were used to explore the best spectral region by the lowest RMSECV. The interval widths of 4 and 16 were also employed to construct the regression model, dividing the spectrum into 32 and 8 subintervals, respectively. Table 3 shows the calibration and validation results of the optimal iPLS models on THz absorption spectra.

**Table 3:** Calibration and validation results obtained with the optimal iPLS regression model.

Interval Variables	Frequency Range	R_cal_	RMSEC	RMSECV
4	0.731–0.956 THz	0.991	0.768	1.260
8	0.787–0.900 THz	0.992	0.573	0.967
16	0.675–1 THz	0.984	0.837	1.237

As shown in Table 3, the iPLS model with eight interval variables has the lowest RMSECV value (0.967), the lowest RMSEC value (0.573), and the highest R value (0.992), compared to those of the full spectrum PLS model (Table 2). It is clear that the performance of the subinterval PLS model was better than the full-spectrum PLS model.

**Figure 6:**
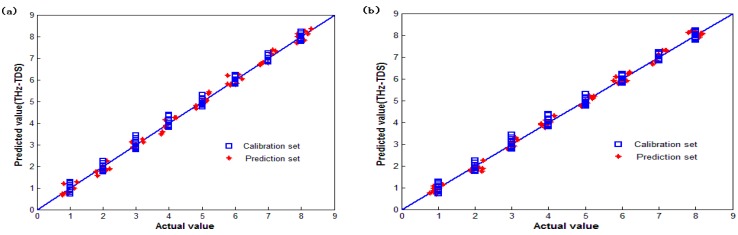
Scatter plots of the actual value *vs.* the predicted value for the discrimination of wheat varieties using (**a**) the full-spectrum PLS calibration model and (**b**) the iPLS calibration model based on the selected subinterval in the range 0.787–0.9 THz.

Figure 6 shows plots of the actual values compared to the values predicted by the PLS models based on the full THz absorption spectra and the optimal interval in the 0.787–0.9 THz range. The RMSEP of the full-spectrum PLS model is 0.845, while the RMSEP of the iPLS model is improved to 0.642.

In Figure 6, the eight subintervals along the X-axis represent the eight wheat varieties as follows: intervals from 0.5–1.5, 1.5–2.5, 2.5–3.5, 3.5–4.5, 4.5–5.5, 5.5–6.5, 6.5–7.5, and 7.5–8.5 represent samples 1–8, respectively. The Y-axis represents the predicted model value for each wheat variety. The maximum range of values predicted by the PLS model for the discrimination wheat grains is 0.67–1.28, while that for the iPLS model is 0.70–1.15, which produces satisfactory results in the evaluation of the wheat varietals. The dispersion degree of the predicted value for Aobiao wheat and Zhengmai 9023 wheat is relatively high, as shown in Figure 6a, but both varieties can be discriminated correctly.

Comparing Figure 6a,b, improved prediction accuracy of the optimized PLS model for the discrimination of wheat varieties is observed, and the relative prediction error in the full-spectrum model is reduced. These results indicate that both the PLS and iPLS models can obtain good predictions for discriminating wheat varieties using THz spectroscopy; however, the iPLS model improves the prediction accuracy through optimal spectral region selection technology.

### 3.4. Discussion

Compared with other spectroscopic methods, such as NIR, MIR, and Raman, THz spectroscopy is a non-destructive rapid method to discriminate wheat varieties. The authors of [6] report that both MIR and NIR techniques can be applied in the industry as a rapid analytical tool to measure the quality parameters. In our experiment, the total time required for preparation, measurement, and analysis of wheat samples was within 5 min, which is also equal to that in [6,7] using data fusion with multiple analytical measurements to increase the model performance and decrease the modeling error for the prediction of quality parameters of crude oil, and the average RMSEP for the fully fused model (which included the IR, Raman, and NMR data) was calculated to be 0.307%. However, few studies compare the iPLS and PLS methods on the spectroscopic data of sample for the improvement in model performance.

Due to complex compositions in the wheat, the proportion of the characteristic components is small, and the overlapping signals obscure the THz absorption spectra. The absorption spectra for the wheat samples obtained in this experiment are featureless. In fact, featureless spectral absorption in the THz range that increases with increasing frequency is expected for many disordered amorphous materials [23,24,25]. Even though the characteristic absorption peaks are lacking, variations in the composition of different wheat samples generate changes in the THz absorption curves. The absorption spectra of various samples have markedly different THz spectra. Thus, chemometric analysis was employed using PLS regression to discriminate the eight wheat samples.

To obtain the best prediction model, both PLS and iPLS analysis methods were applied to construct regression models for discriminating the wheat varieties based on the THz absorption spectra. The above-mentioned analysis indicates that the model performance is primarily affected by the selection of the subinterval range. An appropriate interval width can provide the best prediction results. If the subinterval width is too large, noise and irrelevant information as well as the input sample spectra cause the model to predict the wheat varieties with lower accuracy.

Furthermore, correlations between neighboring regions in the spectrum cannot be obtained by the iPLS model [26]. In particular, the PLS model has only one subinterval (the full spectrum), which includes all spectral information without removing the noise or irrelevant intervals. Thus, the PLS performance is not better than the iPLS model performance. If the interval width is too low, and the corresponding subinterval is too broad, high prediction error will be caused by the loss of small signature information in the spectrum.

Moreover, the prediction accuracy of the constructed model is also affected by the THz absorption of sample. The measured THz spectra of sample are dependent on the experimental environment, experimental setup, background noise, uncertainty of the time-domain signal system [27], and sample preparation process. Moreover, the chemical constituents of wheat grains are determined by many factors [28], such as the wheat variety, plant environment, climate, harvest year, and so on. Even though wheat samples from different harvest years are prepared, the absorption spectra of samples are likely different from each other due to the difference between the chemical compositions of samples. To construct an accurate PLS regression model for wheat varietal discrimination using THz absorption spectra, the influencing factors of the spectra, such as wheat samples from different harvest years in the same plant area and wheat samples from different harvest years and different plant areas, will need to be considered and explored in further systemic studies.

In our work, to avoid the influence of these factors on the performance of the prediction model, the average spectra of the samples were produced by repeated measurements under the same conditions. The PLS regression model was developed to discriminate the wheat varieties based on the THz spectra, and iPLS was used to remove irrelevant information from the spectra. The RMSECV and RMSEC values were used to evaluate the performance of the PLS model. The best subinterval in the iPLS model, which was determined using the lowest RMSECV, was selected for the PLS calibration model. The prediction results for the wheat varieties were improved by using the iPLS model with eight interval variables and the lowest RMSECV value of 0.967.

## 4. Conclusions

In this study, the potential of THz spectra as a new, non-destructive technology for wheat varietal discrimination has been demonstrated. First, the THz spectra from 0.2 to 2 THz were measured and analyzed for eight wheat varieties using the transmission configuration. Then, PLS regression was employed to obtain a prediction model for wheat varieties. For comparison, iPLS was also employed to improve the performance of the calibration model using the proper subinterval in the spectrum. The results showed that good prediction models could be obtained with lower RMSECV and higher R-values in the two regression methods. In particular, the prediction accuracy for wheat grain varieties was improved with the iPLS calibration model (*R* = 0.992 and RMSECV = 0.967). As a result, THz spectroscopy associated with chemometric techniques showed useful measurement and discrimination of wheat varieties. However, further studies should be considered to account for the large number of mixed varieties and for varieties harvested in different years.
